# Functional and biochemical inflammatory responses to a 75 ng intra-articular recombinant equine interleukin-1β (reIL-1β) synovitis model

**DOI:** 10.3389/fvets.2026.1874132

**Published:** 2026-09-15

**Authors:** L. Korac, L. St. George, P. McCrae, J. MacNicol, N. Golestani, J. Souccar-Young, N. Karrow, Á. Cánovas, A. Avison, W. Pearson

**Affiliations:** 1 Department of Animal Biosciences, University of Guelph, Guelph, ON, Canada; 2 School of Health, Social Work and Sport, University of Lancashire, Preston, United Kingdom; 3 Department of Animal Biosciences, Centre for Genetic Improvement of Livestock, University of Guelph, Guelph, ON, Canada; 4 Department of Biomedical Sciences, Ontario Veterinary College, University of Guelph, Guelph, ON, Canada

**Keywords:** equine joint inflammation, inertial measurement units (IMU), objective gait analysis, prostaglandin E_2_, synovial fluid biomarkers

## Abstract

Recently, a synovitis model using 75 ng intra-articular recombinant equine interleukin-1 (reIL 1β), showed promise for inducing a mild, self-limiting inflammatory response without overtly altering upper-body movement asymmetry in *n* = 3 horses. This study aimed to evaluate the effect of this 75 ng reIL-1β model on gait biomechanics and synovial fluid (SF) inflammatory, pro-resolution, and cartilage turnover biomarkers in a larger equine sample. SF samples were collected from the left intercarpal joint of *n* = 11 horses at baseline and at 6- and 12-hours following 75 ng reIL-1β injection and were analyzed for prostaglandin E₂ (PGE₂), nitric oxide (NO), glycosaminoglycan (GAG), chondroitin sulfate 846, and resolvin D1. Prior to each arthrocentesis, horses underwent subjective (AAEP) and objective (inertial measurement unit (IMU)) gait analysis during in-hand trot trials. Upper-body movement asymmetry parameters (MinDiff and MaxDiff) were calculated from poll and pelvis IMU data. Linear mixed models and Spearman correlation coefficient compared biomarker concentration and asymmetry parameters between timepoints. At 6-hours, PGE₂ and NO concentrations and pelvis MinDiff increased significantly compared to baseline (*p* < 0.05). At 12-hours, GAG concentration significantly decreased and poll and pelvis MinDiff significantly increased compared to baseline (*p* < 0.05). Importantly, changes in poll and pelvis movement asymmetry (<7 and ±1.2 mm, respectively) fell within thresholds associated with between-session variability, and changes in intra-articular PGE₂ and NO concentrations were comparable to those observed following mild exercise. These findings provide further evidence to support the use of this ethically acceptable 75 ng reIL-1β model for inducing a low-grade, transient inflammatory response, without overtly altering movement.

## 1 Introduction

Experimental models of equine joint inflammation have proven crucial for advancing our understanding of equine joint inflammatory processes, synovitis, and the pathophysiology of osteoarthritis, a debilitating condition that contributes substantially to economic loss within the equine industry (1–3). These self-limiting experimental models are characterized by the intra-articular administration of inflammatory stimuli (4–6), namely lipopolysaccharides (LPS) (4–7) or recombinant equine interleukin-1β (reIL-1β) (5, 8, 9), which are administered at concentrations that elicit synovial inflammation and resultant in subsequent clinical lameness (5, 7, 10). The mechanism of action for intra-articular LPS models occurs through activation of TLR4, which initiates both MyD88- and TRIF-dependent signaling pathways, resulting in NF-κB activation and production of pro-inflammatory cytokines (11). This produces a robust and broad inflammatory response characterized by rapid increases in synovial inflammatory mediators (11–13). In contrast, intra-articular reIL-1β injection produces a more targeted inflammatory response via IL-1 receptor signaling that is primarily MyD88-dependent, leading to transient activation of NF-κB and MAPK pathways (14–16). Both LPS and reIL-1β models induce synovitis that is characterized by increased SF inflammatory biomarkers, supporting their utility for investigating early joint inflammatory events (5, 9). However intra-articular reIL-1β produces a comparatively more targeted and transient inflammatory response than LPS (5, 10, 17), making it an ethically compatible technique for studying synovitis in equine joints.

In addition to inducing transient increases in SF inflammatory biomarkers, the dose-dependent effects of intra-articular reIL-1β administration on transient clinical lameness are well described in horses (5, 10, 15, 18, 19). As an example, intercarpal injection of 100 ng reIL-1β induces transient, mild to moderate (2–3.5/5 AAEP) clinical forelimb lameness, that is typically observed within 4 h post-injection and peaks between 6 and 8 h (5, 10). This 100 ng reIL-1β dose also elicits marked increases in intercarpal joint SF inflammatory biomarkers, including prostaglandin E₂ (PGE₂) (9), with elevations also observed as early as 4 h post-injection and sustained for up to 24 h (5). Higher doses of 200 ng reIL-1β, administered into the stifle joint, have resulted in moderate to severe hindlimb lameness by 16-h post-injection, alongside synovitis characterized by increased SF white blood cell counts and total protein concentrations (18). Although methodological differences limit direct comparisons across studies, these findings reveal a positive relationship between intra-articular reIL-1β dose and clinical lameness severity and biochemical indicators of joint inflammation (5, 10, 18).

In recognition of the dose-dependent effects of intra-articular reIL-1β administration, our research group conducted pilot work to determine whether comparatively lower-doses of reIL-1β could induce a transient intra-articular inflammatory response without significantly altering gait biomechanics (20). This work demonstrated that intercarpal administration of 50 ng and 75 ng reIL-1β doses resulted in significant increases in SF PGE₂ concentrations, with no concurrent changes in nitric oxide (NO) or glycosaminoglycan (GAG) concentrations. In addition, objective gait analysis was employed to determine whether these inflammatory responses were associated with changes in gait biomechanics. Specifically, inertial measurement units (IMUs) were employed to calculate upper-body movement asymmetry parameters that are increasingly used to objectively inform clinical decision making for lameness evaluations (21–23) and in research for quantifying the effect of various interventions on equine movement (4, 7, 18, 24–27). These asymmetry parameters represent the difference in the two minima (MinDiff) and maxima (MaxDiff) of head and pelvis vertical movement, that occur during the left and right stance phases of one trot stride (28). The MinDiff and MaxDiff are, respectively, associated with asymmetries related to the impact and push-off phase of the gait cycle, with a value of 0 mm indicating perfect symmetry between left and right limbs (28). This integration of objective gait analysis and physiological SF-derived outcome measures notably revealed significant increases in poll MinDiff following 75 ng reIL-1β injection, which was positively correlated with PGE_2_ concentration (20). However, changes in poll MinDiff did not exceed the thresholds associated with inter-session variability for this asymmetry parameter, indicating that functional responses to a 75 ng dose of reIL-1β were likely associated with biological variation (29). Although insightful, this pilot study was limited by a small sample of three horses and the analysis of only three SF inflammatory biomarkers. Thus, further work is justified to elucidate the physiological and functional effects of this 75 ng reIL-1β inflammatory model, by studying additional SF biomarkers in a larger sample of horses.

The SF biomarkers enable researchers to characterize inflammatory and cartilage metabolism responses to experimentally-induced, or clinical synovitis in horses (9, 10, 30, 31, 46). Accordingly, the aforementioned pilot study evaluated SF PGE₂, NO, and GAG biomarker concentrations, which are widely used to assess intra-articular inflammation and cartilage degradation in horses (20, 32–34). The PGE₂ and NO represent inflammatory mediators that are produced by synoviocytes and chondrocytes in response to inflammatory stimuli, and are associated with inflammatory signaling, cartilage matrix degradation, and nociceptive pathways (33, 35, 36). In contrast, increased SF GAG concentration reflects cartilage extracellular matrix turnover (11, 12). While these biomarkers provide insight into inflammatory activation and tissue breakdown, they do not capture pathways involved in cartilage repair or the resolution of inflammation, which are increasingly recognized as important components of joint homeostasis (37–39). Therefore, the present study seeks to expand the SF biomarker panel to include chondroitin sulfate 846 (CS846), an epitope associated with aggrecan synthesis and cartilage matrix turnover (40, 41), and resolvin D1 (RvD1), a specialized pro-resolving lipid mediator involved in the active resolution of inflammation (39, 42, 43). The collective evaluation of these biomarkers that represent inflammatory activation, cartilage metabolism, and inflammatory resolution will facilitate a more comprehensive characterization of the biological responses to intra-articular reIL-1β administration (31, 44). Further, combining SF biomarker concentration with objective gait analysis will provide complementary insight into the physiological and functional mechanisms of joint inflammation that occur following an intra-articular reIL-1β challenge (7, 20). Thus, the aim of this study is to quantify the effects of a 75 ng reIL-1β intra-articular inflammatory model on SF inflammatory mediator and resolution and cartilage metabolism biomarkers, and functional upper-body movement asymmetry parameters, over a 12-h period. Based on our previous pilot work (20), we hypothesized that intra-articular administration of equine 75 ng reIL-1β would induce local joint inflammation, resulting in increased SF biomarker concentrations, without producing changes in upper-body movement asymmetry that exceed inter-session variability ranges (29).

## 2 Materials and methods

The use of horses in this study was reviewed and approved by the University of Guelph Animal Care Committee (AUP #4764), in compliance with the guidelines set forth by the Canadian Council on Animal Care. The experimental methods employed here largely replicated those used in the preceding pilot study (20). Detailed description of the methodology has been described previously by Korac et al. (20).

### 2.1 Animals

Eleven [*n* = 11; size determined based on published work by Pearson et al. (9)] mixed-breed horses (mean ± SD age: 9.1 ± 5.8 years, weight: 503 ± 77.8 kg, sex: 10 mares, 1 gelding) with no clinical history of joint disease, were recruited from the Ontario Ministry of Agriculture, Food and Agribusiness Arkell Equine Research Station (Arkell, Ontario, Canada). Horses were cared for in accordance with the guidelines of the Canadian Council on Animal Care (45). They were provided with water and grass-hay *ad libitum* and were housed in a group-based turnout system. Horses were maintained under this diet and management regimen for a minimum of 12 months prior to study commencement. At the start of the study, all horses were scored as ≤2/5 using the American Association of Equine Practitioners (AAEP) scale, as determined by a qualified veterinarian (A. A.).

### 2.2 Intra-articular challenge

Horses received a single intra-articular injection of 75 ng recombinant equine interleukin-1β (reIL-1β; Kingfisher Biotech, Inc., USA) diluted in 500 μL sterile phosphate-buffered saline and administered into the left intercarpal joint. Consistent with Korac et al. (20), the cytokine was species-specific (*Equus caballus*) and was produced using a *Saccharomyces cerevisiae* expression system (Catalog #RP0757E-005; 10 μg/vial). In accordance with the manufacturer’s instructions, the lyophilized, biologically active protein was reconstituted in sterile phosphate-buffered saline immediately prior to use. Lot-specific biological activity was confirmed using the manufacturer’s Certificate of Analysis. Data collection was performed prior to intercarpal reIL-1β injection (baseline) and at 6- and 12-h post-reIL-1β injection, as described in Section 2.3.

### 2.3 Data collection

#### 2.3.1 Synovial fluid sampling

The method for SF sampling was previously described in Korac et al. (20). Briefly, at baseline, the hair over the left intercarpal joint was clipped, and the site was desensitized using a topical lidocaine/prilocaine formulation (Emla cream; Astra Pharmaceuticals), applied 15 min prior to sampling. The skin was then aseptically prepared with 70% isopropyl alcohol and 4% chlorhexidine solution. A sterile 22-gauge, 1-inch needle was introduced into the intercarpal joint, and approximately 1.5 mL of SF was aspirated into a 3 mL sterile syringe that was immediately transferred into a heparinized 5 mL vacutainer for stabilization. Following aspiration, a separate 3 mL syringe containing 75 ng reIL-1β was attached to the same needle hub and injected slowly into the intercarpal joint space. Additional SF samples were collected using the same protocol at 6- and 12-h post- reIL-1β injection. All SF samples were immediately placed on ice until sampling was complete (≤5 h) and then transported to the laboratory, where they were centrifuged at 5000 × *g* for 15 min. The supernatant was aliquoted into 1.5 mL microcentrifuge tubes containing 10 μg indomethacin to prevent prostaglandin synthesis and stored at −80 °C for subsequent analysis.

#### 2.3.2 Clinical outcomes

Clinical outcomes for this study are reported in Korac et al. (49). Prior to lameness scoring, gait biomechanics assessment, and synovial fluid collection, clinical evaluations were performed for all horses (*n* = 11). Rectal temperature was measured using a digital veterinary thermometer, respiratory rate was determined by counting thoracic excursions (breaths/min), and carpal joint circumference was measured using a flexible measuring tape positioned circumferentially around the injected joint at a consistent anatomical landmark. These assessments were performed at each experimental time point to monitor systemic and local clinical responses following intra-articular reIL-1β administration. No significant changes were observed in any clinical outcome measures across the study time points (all *p* > 0.05).

#### 2.3.3 Gait biomechanics

Subjective and objective gait analysis were conducted prior to SF sampling at each timepoint, using the methods described in Korac et al. (20). Subjective gait assessment was initially conducted by a qualified veterinarian (A. A.) using the AAEP lameness scale. Horses were then equipped with IMU sensors (Avanti, Delsys Inc., USA), attached over the poll and between the tuber sacrale using a combination of Delsys Adhesive Surface Interfaces (Delsys Inc., USA) and double-sided carpet tape. IMU data (194 Hz) were collected from each horse during in-hand trot trials using EMGWorks Software (Delsys Inc., USA. Version 4.8.0). At each timepoint, horses completed four in-hand trot trials, twice in each direction, on a 210 ft. concrete runway. Horses were permitted to trot at their self-selected speed without encouragement from the handler. A smartphone GPS system was mounted on a Polar Equine H10 heart rate strap (Polar Equine, Finland), and measured trot speed through the Polar Flow app, which recorded position-based velocity (km/h) at one-second intervals.

### 2.4 Data analysis

#### 2.4.1 Synovial fluid biomarker analysis

All chemicals and reagents were sourced from Sigma Aldrich (Mississauga, Ontario, Canada), unless otherwise specified. Absorbance measurements for all assays were obtained using a Victor 1,420 Microplate Reader (Perkin Elmer, Woodbridge, ON). The SF samples were thawed to room temperature prior to analysis. PGE₂, NO, and GAG concentrations in SF aliquots were quantified using the methods described by Korac et al. (20) and are concisely descried here. The PGE₂ concentrations were measured using a multi-species ELISA kit (Arbor Assays, Mississauga, ON, Canada; CAT# K051-H5), with samples run in duplicate on antibody-coated 96-well plates and concentrations calculated from fourth-order polynomial standard curves (*R*^2^ > 0.99). NO concentrations were determined using the Griess reaction, where undiluted SF samples were analyzed in duplicate following the addition of sulfanilamide (0.01 g/mL) and N-(1)-naphthylethylenediamine hydrochloride (1 mg/mL) in phosphoric acid (0.85 g/L). The GAG concentrations were quantified using a 1,9-dimethylmethylene blue (DMMB) spectrophotometric assay, in which SF samples were diluted (2:13) in dilution buffer and analyzed in triplicate following the addition of guanidine hydrochloride and DMMB reagent. Absorbance was measured at 450 nm for PGE₂ and 530 nm for NO and GAG assays. Analyte concentrations were calculated from plate-specific standard curves using assay-appropriate regression models: fourth-order polynomial regression for the PGE₂ ELISA (*R*^2^ > 0.99) and linear regression for NO (*R*^2^ > 0.99) and GAG assays (*R*^2^ ≥ 0.98).

The RvD1 concentrations in SF samples were quantified using an equine-specific quantitative ELISA kit (AffiGen Life Science Supply Chain, Bailey’s Harbor, WI; Catalog # AFG-SB-7816). Samples were loaded in duplicate (10 μL), along with 40 μL sample diluent, into antibody-coated wells of a 96-well microtiter plate, as per the manufacturer’s protocol. After adding 100 μL of the HRP-conjugate to all wells, plates were incubated at 37 °C for 60 min. Wells were washed five times (400 μL wash buffer per wash), followed by the addition of 50 μL chromogen A and 50 μL chromogen B to each well. Plates were incubated for 15 min at 37 °C in the dark, after which 50 μL stop solution was added to terminate the color reaction. The optical density was measured at 450 nm using a microplate reader within 15 min of adding the stop solution. A standard curve of optical density versus RvD1 concentration was constructed, and sample concentrations were interpolated from this curve.

The CS846 was quantified in tissue culture media using a competitive ELISA kit (IBEX Pharmaceuticals, Montreal, QC; Catalog #60-1004). The SF samples were diluted 1:5 in the manufacturer’s sample diluent prior to assay. Diluted samples and calibration standards were loaded in duplicate into antibody-coated 96-well microtiter plates according to the manufacturer’s protocol. The competitive assay format employed a monoclonal antibody specific to the CS846 epitope. After incubation with samples and standards, the bound antibody was detected with an HRP-conjugated secondary antibody. Plates were washed thoroughly to remove unbound reagents, developed with substrate solution, and stopped after sufficient color development. Absorbance was measured at 450 nm using a microplate reader. For each plate, a standard curve was generated from the optical density of the calibration standards, and sample concentrations were interpolated from this curve. Blank-corrected values were used in all calculations.

#### 2.4.2 Gait biomechanics

Upper-body movement asymmetry parameters were calculated from IMU data, using the same methods described in the pilot study (20). Briefly, vertical acceleration signals from the poll and pelvis were double-integrated to obtain vertical displacement, with the mean removed after each integration (47). Displacement data were high-pass filtered (Butterworth 4th order), using a cut-off frequency adjusted to 70% of the stride frequency of each horse (48) and then low-pass filtered (Butterworth 4th order) using a 30 Hz cut-off frequency (49). Vertical displacement signals were stride segmented based on pelvis vertical movement maxima (50) and then poll and pelvis MinDiff and MaxDiff were calculated for each stride according to the methods described by Rhodin et al. (51). Outliers were identified in accordance with the methods described by Persson-Sjodin (52). Average trot speed per trial was calculated by averaging GPS-derived stride velocity (km/h) across the trial duration, and converting to m/s.

## 3 Statistical analysis

Statistical analyses were conducted using RStudio (RStudio, USA, version 2024.09.0). To improve statistical power, asymmetry parameters (MinDiff and MaxDiff) from the poll and pelvis were multiplied by −1, resulting in absolute values and asymmetry independent of sidedness. Linear mixed models (LMMs) were used to evaluate the effect of intra-articular reIL-1β challenge over time on trot speed, poll and pelvis asymmetry parameters (MinDiff and MaxDiff), and SF biomarker concentrations (PGE₂, NO, GAG, CS846, and RvD1). Timepoint (hour) was included as a fixed effect, and horse was included as a random effect to account for repeated measures within individuals. To account for inter-horse variation and in accordance with other studies (27, 53–55), the difference in absolute asymmetry (mm) represented the outcome variable for LMM analysis and was calculated within-horse as the difference between the baseline value and the associated values at 6- and 12-h timepoints. Models were fit using the lmer function in the lme4 package, and *p*-values for fixed effects were obtained using Satterthwaite’s approximation via the lmerTest package (56). Estimated marginal means (EMMs) were generated using the emmeans package, and pairwise contrasts were performed to compare each post-injection timepoint (6- and 12-h) with baseline. Spearman’s rank correlation coefficients (*ρ*) were used to evaluate potential correlation between biochemical (SF biomarker concentrations) and functional (asymmetry parameters) outcome measures that were identified as being statistically significant between timepoints using LMM (57). Statistical significance was defined as *p* < 0.05.

## 4 Results

### 4.1 Synovial fluid biomarker concentration

Compared to baseline, PGE₂ and NO concentrations increased significantly at the 6-h timepoint (*p* < 0.01; Table 1) but were non-significant at 12-h (*p* > 0.05; Table 1). Conversely, GAG concentration significantly decreased at the 12-h timepoint but was non-significant at the 6-h timepoint when compared to baseline (*p* < 0.01; Table 1). The CS846 and RvD1 concentrations were not significantly different across any timepoints (*p* > 0.05; Table 1).

**Table 1:** Estimated marginal (EM) mean (± SE) concentrations of prostaglandin E_2_ (PGE_2_; pg/mL), nitric oxide (NO; μM), and glycosaminoglycans (GAG; μg/mL), chondroitin sulfate 846 epitope (CS846; ng/mL), equine resolvin D1 (RvD1; pg/mL) in synovial fluid collected from the intercarpal joints of eleven horses at baseline (0 h) and at 6-, and 12-h following intra-articular injection 75 ng recombinant equine IL-1β (reIL-1β).

Biomarker	Timepoint (h)	EM Mean ± SE	*p*-value
PGE₂	0	55.59 ± 49.54	–
	**6**	**258.28 ± 46.34**	**<0.01**
	12	112.01 ± 53.51	0.65
NO	0	3.40 ± 0.54	–
	**6**	**6.29 ± 0.64**	**<0.01**
	12	4.78 ± 0.69	0.21
GAG	0	7.94 ± 0.46	–
	6	6.73 ± 0.45	0.12
	**12**	**5.38 ± 0.47**	**<0.01**
CS846	0	1211.16 ± 53.46	–
	6	1293.82 ± 68.78	0.54
	12	1280.36 ± 71.28	0.65
RvD1	0	8.60 ± 0.18	–
	6	8.60 ± 0.19	0.99
	12	8.71 ± 0.20	0.88

*p*-values indicate differences between baseline (0 h) and 6-, and 12-h timepoints as determined by linear mixed model analysis. Significant differences are indicated at *p* < 0.05.

### 4.2 Gait biomechanics

Across timepoints, six (*n* = 6) horses received the same subjective AAEP score, while five (n = 5) horses received scores that differed by 1-scale point (between 0 and 1/5). Compared to baseline values, trot speed (m/s) and poll MinDiff (mm) were non-significant at 6-h but significantly increased at the 12-h timepoint (*p* < 0.01; Table 2). Poll MaxDiff values were non-significant across timepoints when compared to baseline (*p* > 0.05; Table 2). Significant increases in pelvis MinDiff were observed at 6- and 12-h timepoints compared to baseline (*p* < 0.05; Table 2). In contrast, significant decreases in pelvis MaxDiff were observed at 6-h compared to baseline (*p* < 0.05; Table 2).

**Table 2:** Estimated marginal (EM) means difference (± SE) and associated *p*-values for poll and pelvis asymmetry parameters (MinDiff and MaxDiff; mm) and trot speed (m/s) between baseline and at 6- and 12-h after injection of 75 ng recombinant equine IL-1β (reIL-1β) into the left intercarpal joint of eleven horses.

Timepoint	Poll MinDiff		Poll MaxDiff		Pelvis MinDiff		Pelvis MaxDiff		Trot speed	
	EM Mean ± SE	*p*-value	EM Mean ± SE	*p*-value	EM Mean ± SE	*p*-value	EM Mean ± SE	*p*-value	EM Mean ± SE	*p*-value
6 h	−0.90 ± 1.39	0.73	−1.03 ± 1.06	0.52	**0.90 ± 0.39**	**0.04**	**−1.17 ± 0.42**	**0.01**	0.05 ± 0.03	0.08
12 h	**6.82 ± 1.42**	**<0.01**	−1.41 ± 1.08	0.33	**0.98 ± 0.39**	**0.02**	−0.51 ± 0.42	0.37	**0.24 ± 0.03**	**<0.01**

Significant differences are indicated at *p* < 0.05.

### 4.3 Correlation between biomarkers and gait biomechanics

Correlations between specific SF biomarker concentrations (PGE₂, NO and GAG) and movement asymmetry parameters (poll MinDiff, pelvis MinDiff and MaxDiff) were evaluated based on the significant effect (*p* < 0.05) of reIL-1β injection on these variables, as determined using LMM. Pelvis MaxDiff and was negatively correlated with SF GAG concentration (*ρ* − 0.33, *p* < 0.01; Table 3) and positively correlated with PGE₂ SF concentration (*p* < 0.01; Table 3). Significant correlations were not observed between the remaining asymmetry and biomarker parameters (*p* > 0.05, Table 3).

**Table 3:** Spearman correlation coefficients (*ρ*) between prostaglandin E_2_ (PGE_2_; pg/mL), nitric oxide (NO; μM), and glycosaminoglycans (GAG; μg/mL) and absolute poll (MaxDiff; mm) and pelvis asymmetry parameters (MaxDiff and MinDiff; mm) from eleven horses following intra-articular injection 75 ng recombinant equine IL-1β (reIL-1β) into the left intercarpal joint.

Biochemical marker	Region	Asymmetry measure	Spearman *ρ*	*p*-value
PGE₂	**Pelvis**	**MaxDiff**	**0.41**	**<0.01**
		MinDiff	−0.07	0.61
	Poll	MinDiff	0.21	0.11
NO	Pelvis	MaxDiff	0.14	0.19
		MinDiff	0.14	0.21
	Poll	MinDiff	0.05	0.63
GAG	Pelvis	**MaxDiff**	**−0.33**	**<0.01**
		MinDiff	0.09	0.46
	Poll	MinDiff	0.08	0.52

## 5 Discussion

A recent pilot study demonstrated promising efficacy of a low-dose reIL-1β model for inducing joint inflammation in a small sample of three horses (20). In the present study, we aimed to further evaluate the effect of this 75 ng reIL-1β model on gait biomechanics (MinDiff, MaxDiff) and SF inflammatory (PGE₂ and NO), pro-resolution (RvD1), and cartilage turnover (GAG) and regeneration (CS846) biomarkers in a larger equine sample (*n* = 11) over 12 h. At 6-h post-injection, SF PGE₂ and NO concentrations and pelvis MinDiff significantly increased, while pelvis MaxDiff significantly decreased, when compared to baseline. At 12-h post-injection, pelvis MinDiff remained significantly greater than baseline, alongside a significant increase in poll MinDiff and decrease in SF GAG concentration. Importantly, the observed changes in movement asymmetry parameters between timepoints were within ±1.2 mm for the pelvis and <7 mm for the poll, which fall within previously reported ranges of between-session variation in healthy horses (29). Together these findings support the hypothesis that intercarpal injection of 75 ng reIL-1β can induce local joint inflammatory processes that result in elevated SF inflammatory mediator biomarkers, without associated changes in upper-body movement asymmetry that exceed inter-session variability ranges. RvD1 and CS846 concentrations were not significantly different across timepoints, and significant decreases in GAG concentration were observed, which does not support the hypothesis that that intercarpal injection of 75 ng reIL-1β induces elevated SF inflammatory resolution and cartilage metabolism and regeneration biomarkers.

PGE₂ is an inflammatory mediator that is upregulated in response to reIL-1β stimulation in equine articular tissue (58–60). Reported SF PGE₂ concentrations in horses range from approximately 100 pg/mL in healthy joints (61) to as high as 2,200 pg/mL in cases of joint disease (9, 32). Further, elevated PGE_2_ concentrations ranging from ~100 to 360 pg/mL (20) and 500 to >2000 pg/mL (9) are reported following intercarpal injection of 75 ng and 100 ng reIL-1β, respectively (5, 17). Significantly elevated PGE2 concentrations at 6-h post-injection fell between reported ranges for healthy and pathological equine joints (REFS), and closely approximate concentrations associated with exercise-induced transient inflammation (~263 pg/mL) (62). These findings suggest that this 75-ng reIL-1β model is effective for inducing a subclinical inflammatory response at the level of the joint, characterized by physiologically relevant elevations in PGE₂ that are consistent with mild, transient inflammation rather than overt pathology. Importantly, peak PGE_2_ concentrations occurred at 6-h post 75 ng reIL-1β injection and returned to a level that did not significantly differ from baseline concentrations by 12 h. This finding contrasts other studies that reported sustained increases in PGE_2_ concentration at 12- and 24-h following inter-carpal injection of 100 ng reIL-1β (5, 17). Thus, the comparatively faster decreases in PGE₂ concentrations observed in the present study may reflect a more rapid resolution of the transient inflammatory response to intra-articular administration of 75 ng reIL-1β (17). Interestingly, relatively lower and more variable PGE₂ concentrations were observed across timepoints when compared to findings from the associated pilot study (20). These discrepancies could be explained by the larger sample size employed in the present study, which may more accurately represent inter-individual inflammatory response mechanisms across the wider equine population. However, further studies are required to confirm this interpretation.

Like PGE₂, SF NO concentrations significantly increased at 6-h following intra-articular administration of 75 ng reIL-1β and returned to concentrations that did not significantly differ from baseline by 12-h. Equine osteoarthritic joint tissues, including synovial membrane and articular cartilage, produce elevated NO, which supports its role in mediating inflammatory and catabolic processes within the joint (35). Unfortunately, SF NO concentrations demonstrate considerable variability and overlap between healthy and diseased equine joints (33), which limits its usefulness for distinguishing equine joint disease (33). Significant increases in SF NO concentrations at 6-h post-injection fell within reported ranges for healthy equine joints (~5–35 μM) (33, 61) and following an acute exercise bout (~2–11 μM) (62). This suggests that, while the 75 ng reIL-1β model is sufficient to induce an inflammatory response associated with early ECM turnover, the magnitude of NO production is more consistent with a controlled, physiological response rather than the pathological response that is characteristic of osteoarthritis (61, 62). Like PGE₂, some discrepancies between the present study and associated pilot were observed (20). Notably across time points, NO concentrations were comparatively lower and less variable than the pilot study, which likely contributed to the detection of a significant effect at 6-h, whereas greater variation in the pilot study may have limited statistical power (20). Still, findings from both studies demonstrate that this 75 ng reIL-1β model elicits increased SF NO concentration, but at levels that are indicative of a transient physiological response.

The GAGs are negatively charged polysaccharides that are primarily derived from cartilage ECM proteoglycans, and are thus commonly used as biomarkers of ECM turnover within the joint (9, 34, 44). Because ECM degradation is a hallmark of osteoarthritis, the release of GAGs into SF is generally interpreted as an indicator of proteoglycan loss associated with cartilage catabolism (34, 63). When measured using DMMB-based assays, GAG concentrations from equine intercarpal SF vary markedly across studies, ranging from approximately 20 μg/mL in clinically healthy horses (9) to >250 μg/mL in horses with joint pathology (9, 63). A study showed that intercarpal injection of 100 ng of reIL-1β significantly increased equine SF GAG concentrations to ~75 μg/mL 24-h post-injection (9). In the present study, SF GAG concentrations, also derived from DMMB-based assays, fell within reported ranges for healthy joints (9) across timepoints. Interestingly, GAG concentrations decreased following intra-articular administration of 75 ng reIL-1β and were significantly lower than baseline at the 12-h timepoint. However, it is important to note that the magnitude of change was small, with decreases of less than 1.5 μg/mL between consecutive 6-h timepoints, which is unlikely to represent a biologically meaningful reduction in cartilage turnover. This finding differed from the pilot study, where GAG concentrations did not change significantly across timepoints, despite higher EM mean values and a greater degree of change than observed here (20). These discrepancies may be explained by methodological differences, including sample size and the number of repeated arthrocentesis performed, which is known to affect GAG concentration in equine SF (20, 61). Collectively, these findings suggest that the 75 ng reIL-1β challenge did not produce sufficient ECM catabolism over the 12-h sampling period to elicit a robust proteoglycan release response (64). Future studies would benefit from incorporating additional markers of cartilage structural damage, such as cartilage oligomeric matrix protein, to better distinguish between subtle changes in matrix turnover and true cartilage breakdown (65).

The transient nature of the 75 ng reIL-1β model studied here provides an opportunity to assess inflammatory resolution processes and the subsequent progression of transient synovitis. Thus, the rationale for investigating SF biomarkers of aggrecan turnover (CS846) and inflammation resolution (RvD1) was to extend evaluation beyond the initial phases of inflammation. RvD1 is an omega-3-derived specialized pro-resolving mediator that acts to terminate inflammation (43, 66), promote clearance of inflammatory cells, and restore joint homeostasis (11, 41, 42). The CS846 is a sulfation epitope expressed on newly synthesized chondroitin sulfate chains and is commonly used as a biomarker of cartilage metabolism and aggrecan turnover (67–69). Together, these measures provide insight into the balance between ECM degradation, synthesis and inflammation resolution in response to joint injury or synovitis (69–71). Interestingly, both CS846 and RvD1 concentrations were comparable across the 12-h sampling period and did not significantly differ from the baseline condition. Concentrations of RvD1 in healthy equine SF range from ~6.5–7.0 pg/mL and increase to ~7.5 pg/mL following mild exercise (38). The SF RvD1 concentrations observed in the present study are comparable to values reported in response to mild exercise (38). In healthy equine joints, SF CS846 concentrations are reported to be ~3,000 ng/mL, increasing to ~5,000 ng/mL in osteoarthritic joints (44). Here, CS846 concentrations at each measured timepoint reflected those observed in healthy equine SF (44). These findings suggest that intercarpal injection of 75 ng reIL-1β was not sufficient to increase CS846 and RvD1 concentrations over a 12-h sampling period beyond values reported for healthy equine SF, which further supports the mild, transient nature of the inflammatory response that is induced by this model (38, 44). However, further studies should explore longer sampling durations and other biomarkers with the same function to confirm this interpretation.

The asymmetry parameters evaluated in this study indirectly reflect differences in vertical loading (MinDiff) and propulsive (MaxDiff) forces between left and right fore- and hindlimb pairs (28, 72, 73). In the present study, poll MinDiff was not significantly different from baseline at 6-h post reIL-1β injection but significantly increased at 12-h. Importantly, the magnitude of this change (<7 mm) at 12-h remained within the range of reported inter-session variability in clinically healthy horses (29, 74). In the pilot study, poll MinDiff increased significantly at both 6- and 12-h post-injection (20), with the degree of change also falling within inter-session variability ranges at both timepoints (29, 74). Additionally, poll MaxDiff remained non-significant across timepoints, which is consistent with findings from the pilot study (20). Pelvis MinDiff significantly increased at 6- and 12-h post-injection and pelvis MaxDiff significantly decreased at 6-h. However, across timepoints the magnitude of change (<1.5 mm) and variation (<0.5 mm) for pelvis asymmetry parameters was minimal. Like poll MinDiff, the degree of change for pelvis asymmetry parameters fell within ranges of between-session variability in clinically healthy horses (29, 74). Changes in pelvis asymmetry parameters differed from the pilot study, where MinDiff and MaxDiff significantly decreased at 12-h following 75 ng reIL-1β injection. However, the magnitude of these changes (<4 mm) also fell within inter-session variability ranges (29, 74). These results suggest that the minor, albeit significant, changes in poll and pelvis asymmetry are unlikely to reflect meaningful compensatory adaptations in fore- or hindlimb loading and instead represent normal fluctuations in locomotor symmetry between measurement sessions (29, 74). Accordingly, any associations between SF biomarkers and upper-body asymmetry parameters observed here, and in the pilot, should be interpreted cautiously, as the limited magnitude of biomechanical change reduces the likelihood of detecting robust relationships. These results, combined with the lack of change in clinical lameness scores across timepoints, suggest that the 75 ng reIL-1β model induced only subtle and transient functional changes in vertical head and pelvic movement, that are indicative of biological, inter-session variability (29, 74) and not with intercarpal joint inflammatory response mechanisms.

## 6 Conclusion

This study demonstrates that intra-articular administration of 75 ng reIL-1β induces a transient and mild inflammatory response within the intercarpal joint, characterized by increases in synovial fluid PGE₂ and NO at 6-h post-injection. Despite statistical significance, the magnitude of these biochemical changes was comparable to intra-articular PGE₂ and NO concentrations observed in healthy horses and following mild exercise bouts. Similarly, any significant changes in upper-body movement asymmetry parameters fell within established ranges of between-session variability, suggesting that intra-articular administration of 75 ng reIL-1β is insufficient to elicit functional adaptations. Taken together, the biochemical and biomechanical outcome measures indicate that this equine synovitis model elicits a controlled, low-grade, and transient (<12-h) inflammatory response without objective gait alterations beyond accepted ranges of between-session variability and with no clear evidence of altered cartilage metabolism within the 12-h observation period. These findings support the use of a 75 ng intra-articular reIL-1β challenge as an ethically refined model for investigating early inflammatory processes in equine joints.

### 6.1 Limitations

This study is not without limitations that should be considered when interpreting its findings. The absence of a sham-injected control group represents a limitation of the present study, as it precludes complete differentiation between responses induced by reIL-1β administration and those potentially associated with repeated arthrocentesis, handling, or other procedural factors. The sample size was relatively small (*n* = 11), which may limit the generalizability of the results to the broader equine population. Additionally, the study was conducted under controlled conditions that may not fully reflect real-world training or competition environments, where variables such as surface type, workload intensity, and environmental factors can influence both gait mechanics and inflammatory responses (75–78, 83, 84). Furthermore, trot speed was not standardized across horses or timepoints, which is known to affect objective gait parameters (79–81). The study design also incorporated a limited number of measured time points, which may have restricted the ability to fully characterize the temporal profile of inflammatory responses (62). Similarly, only a select panel of SF biomarkers were evaluated, which may not comprehensively represent the complexity of joint inflammation and tissue metabolism (14, 82). Future studies should build on this work by incorporating larger and more diverse equine sample, increasing the number of measured time points, and evaluating a broader panel of biochemical and molecular markers, which could provide comprehensive insight into the relationship between low-grade joint inflammation and movement asymmetry in horses.

## Data Availability

The original contributions presented in the study are included in the article/supplementary material, further inquiries can be directed to the corresponding author.
